# Initiation of an Inflammatory Response in Resident Intestinal Lamina Propria Cells -Use of a Human Organ Culture Model

**DOI:** 10.1371/journal.pone.0097780

**Published:** 2014-05-19

**Authors:** Jutta Schröder-Braunstein, Judith Gras, Benedikt Brors, Sonja Schwarz, Timea Szikszai, Felix Lasitschka, Guido Wabnitz, Antje Heidtmann, Young-Seon Lee, Serin Schiessling, Christine Leowardi, Mohammed Al-Saeedi, Alexis Ulrich, Antonia Engelke, Johannes Winter, Yvonne Samstag, Thomas Giese, Stefan Meuer

**Affiliations:** 1 Institute of Immunology, University Hospital Heidelberg, Heidelberg, Germany; 2 Division of Theoretical Bioinformatics, German Cancer Research Center (DKFZ), Heidelberg, Germany; 3 Institute of Pathology, University Hospital Heidelberg, Heidelberg, Germany; 4 Department of Surgery, University Hospital Heidelberg, Heidelberg, Germany; 5 Department of Anesthesiology, University Hospital Heidelberg, Heidelberg, Germany; 6 Department of Surgery, Salem Hospital, Heidelberg, Germany; 7 Department of Surgery, St. Vincentius Hospital, Speyer, Germany; 8 Department of Gastroenterology, Hepatology, and Endocrinology, Hannover Medical School, Hannover, Germany; Duke University Medical Center, United States of America

## Abstract

Resident human lamina propria immune cells serve as powerful effectors in host defense. Molecular events associated with the initiation of an intestinal inflammatory response in these cells are largely unknown. Here, we aimed to characterize phenotypic and functional changes induced in these cells at the onset of intestinal inflammation using a human intestinal organ culture model. In this model, healthy human colonic mucosa was depleted of epithelial cells by EDTA treatment. Following loss of the epithelial layer, expression of the inflammatory mediators *IL1B, IL6, IL8, IL23A, TNFA, CXCL2*, and the surface receptors CD14, TLR2, CD86, CD54 was rapidly induced in resident lamina propria cells *in situ* as determined by qRT-PCR and immunohistology. Gene microarray analysis of lamina propria cells obtained by laser-capture microdissection provided an overview of global changes in gene expression occurring during the initiation of an intestinal inflammatory response in these cells. Bioinformatic analysis gave insight into signalling pathways mediating this inflammatory response. Furthermore, comparison with published microarray datasets of inflamed mucosa *in vivo* (ulcerative colitis) revealed a significant overlap of differentially regulated genes underlining the *in vivo* relevance of the organ culture model. Furthermore, genes never been previously associated with intestinal inflammation were identified using this model. The organ culture model characterized may be useful to study molecular mechanisms underlying the initiation of an intestinal inflammatory response in normal mucosa as well as potential alterations of this response in inflammatory bowel disease.

## Introduction

Investigations aimed at understanding molecular events underlying intestinal inflammation are largely focused on chronic processes, summarized as inflammatory bowel diseases (IBD). Moreover, such studies are mostly performed in symptomatic, i.e. ongoing pathological situations: in the murine system following treatment with artificial agents such as dextran sulfate (DSS) [1]; in humans, tissues derived from inflamed intestine in Crohn’s disease, ulcerative colitis etc. Needless to say, experiments with human material are rather scarce when compared to investigations in experimental animal models. Given, therefore, that so far only symptomatic disease situations have been studied, it seems not surprising that all established therapies to treat IBD are of symptomatic nature [2], [3].

As yet, virtually nothing is known concerning the conditions driving intestinal inflammation at its very onset. The central and obvious question, namely, how resident immune effector cells of the intestine differentiate from their physiologic quiescent stages into activated, inflammation producing cell types remains unanswered.

In this regard, previous data published by others and ourselves employing human mucosal tissues virtually exclude the possibility that intestinal T lymphocytes, one major effector population in inflammation, are, under physiological conditions, able to mount adaptive immune responses since their T cell receptor (TCR) reactivities are strongly inhibited by a number of local mechanisms [4]–[9]. Furthermore, innate recognition of commensal microbes is restricted by low or absent expression of pattern recognition receptors (PRR), like CD14, TLR4, and PRR-associated signalling molecules in lamina propria myeloid cells (LPMO) [10]–[13].

Here, we make an attempt to address the onset of intestinal inflammation in a standardized organ culture model employing healthy human colonic tissues. Once exposed to ethylenediaminetetraacetic acid (EDTA), which leads to the detachment of the luminal epithelial layer [14], resident lamina propria cells undergo a vigorous activation process. The present study describes their phenotypical and functional changes at a molecular level. We propose that this model has the potential to collect important information on those molecular mechanisms that drive the initiation of intestinal inflammation.

## Materials and Methods

### Tissue Samples

Gut specimens were derived from individuals undergoing resection for localized colon cancer or benign colonic diseases (e.g. diverticular disease). Microscopically normal colonic mucosa was dissected from the surgical specimen near the resection margin and immediately subjected to the experimental procedures.

For analysis of *DUSP2* gene expression in normal and inflamed intestinal tissue, transmural gut specimens (snap frozen in liquid nitrogen immediately after surgical resection) were provided by the Institute of Pathology, University Hospital Heidelberg. Inflamed gut specimens were derived from patients suffering from ulcerative colitis (UC) or Crohn’s disease (CD). The diagnosis was confirmed by histopathological and clinical criteria prior to qRT-PCR analysis. Specifically, samples considered to be inflamed were characterized by dense mononuclear cell infiltrations, crypt abscesses/cryptitis, erosions or ulcerations. Normal control tissue was obtained from individuals suffering from localised colon cancer or benign colonic disease.

### Antibodies

For immunoflourescent staining of tissue sections the following primary antibodies were employed: CD68, 1 µg/ml (clone: PGM-1, Dako Cytomation, Hamburg, Germany); CD14, 1 µg/ml (clone: TÜK4, Dako); CD86, 10 µg/ml (clone: FUN-1, BD Pharmingen, Heidelberg, Germany). Isotype- and concentration-matched control antibodies (Dako) served as negative controls.

For flow cytometric analysis the following mAb were purchased from BD Bioscience (Heidelberg, Germany): CD3 V450, CD14 FITC, CD33 PE-Cy7, CD86 AF700, HLA-DR V500, CD117 APC.

### “Loss of Epithelial Layer” (LEL) Organ Culture

Mucosal specimens were washed extensively in RPMI 1640 (Life Technologies, Paisley, UK) containing antibiotics prior to removing the mucus layer by dithiothreitol (DTT) treatment (1 mM, 10 min; Sigma, St. Louis, MO, Germany). Subsequently, punches of defined surface area were prepared and denuded of epithelial cells by exposure to 0.7 mM EDTA (Sigma) in HBSS (without Ca^2+^ and Mg^2+^; Life Technologies) at 37°C in a shaking water bath for 30 min (50 ml EDTA/HBSS per punch) [14]. This incubation was repeated three times with two washing steps (10 min in HBSS/antibiotics) after each incubation period.

Where indicated, total mucosa specimens of the same surface area were cultured under identical conditions.

### Isolation of Lamina Propria and Peripheral Blood Leukocytes

Lamina propria leukocytes (LPL) were isolated by enzymatic tissue digestion according to a modified method of Bull and Bookman [14]. Briefly, after depletion of the epithelial layer (see above) mucosal tissue was cut into 2–4 mm pieces and digested in a shaking waterbath at 37°C for 1.5 h using collagenase IV (70 µg/ml; Sigma) and deoxyribonuclease I (100 µg/ml; Sigma) in RPMI 1640 containing 2% fetal calf serum (Sigma), 2% L-glutamine (Life Technologies), and antibiotics. The resulting cell suspension was separated from undigested tissue by filtration through a 70 µm nylon mesh (BD Bioscience, Heidelberg, Germany). For further purification, the cell suspension was subjected to Percoll (GE Healthcare, Munich, Germany) and Ficoll-Hypaque (GE Healthcare) density gradient centrifugation.

Peripheral blood was taken during the operation. Peripheral blood mononuclear cells (PBL) were obtained by Ficoll-Hypaque (GE Healthcare) density gradient centrifugation.

### Gene Expression Analysis by qRT-PCR

Tissue samples were disrupted with the aid of a RiboLyser device (ThermoHYBAID, Heidelberg, Germany) in lysing matrix “D” tubes (Q-BIOgen, Heidelberg, Germany) containing 400 µl lysis buffer from the MagnaPure mRNA Isolation Kit II (ROCHE Diagnostics, Mannheim, Germany). 300 µl of the lysate was collected and mixed with 600 µl capture buffer containing oligo-dT. After centrifugation at 13000 rpm for 5 min, 880 µl of this mix was transferred into a MagnaPure sample cartridge and mRNA was isolated with the MagnaPure-LC device using the mRNA-II standard protocol. MRNA was reverse transcribed using AMV-RT and oligo- (dT) as primer (First Strand cDNA synthesis kit, Roche) according to the manufactures protocol. Primer sets optimized for the LightCycler (RAS, Mannheim, Germany) were developed and provided by SEARCH-LC GmbH (Heidelberg, Germany). The qRT-PCR was performed with the LightCycler FastStart DNA Syber Green I kit (RAS) according to the protocol provided in the parameter specific kits. To correct for differences in the content of mRNA, the calculated transcript numbers were normalized according to the expression of the housekeeping gene peptidylprolyl isomerase B (*PPIB*). Values were thus given as transcripts per 1000 transcripts of *PPIB*.

### Double Immunofluorescence Staining of Tissue Sections

Staining was performed on 2 µm sections of formalin fixed, paraffin embedded human colon tissue. After deparaffinization in graded alcohols, heat induced antigen-retrieval was achieved by incubating the slides in a pressure cooker for 5 min in citrate buffer, pH 6.0. Double immunofluorescence staining was performed using a combination of CD68 (mouse mAb, IgG3) and either CD14 (mouse mAb, IgG2a) or CD86 (mouse mAb, IgG1κ). Incubations were performed overnight at 4°C in antibody diluent (DCS, Hamburg, Germany). Biotinylated rabbit anti-mouse IgG1, (2 µg/ml; Zymed, San Francisco, CA, USA), biotinylated rabbit anti-mouse IgG2a (4 µg/ml; Zymed) and sheep anti-mouse IgG3 (1∶100, lot number AAM05; AbDserotec, Oxford, UK) served as secondary antibodies (30 min at room temperature). DyLight488-conjugated donkey anti-sheep antibody (green fluorescence; 8 µg/ml; Dianova), and Cy3-conjugated streptavidin (red fluorescence; 2 µg/ml; Dianova) were used as secondary reagents (30 min at room temperature). Slides were mounted and viewed with a TCS SL confocal microscope (Leica Microsystems, Wetzlar, Germany).

For quantification, CD14^+^ and CD86^+^ cells were counted in an area (lamina propria) defined by 100 CD68^+^ cells. Three different areas were evaluated per experiment. In order to allow for consistent and comparable results within/between the different experimental conditions areas densely populated by CD68^+^ subepithelial macrophages were excluded from the analysis.

### Flow Cytometry

1×10^5^ to 1×10^6^ cells were incubated with a cocktail of up to six antibodies labelled with different fluorochromes. Peripheral blood monocytes (PBMO) were identified as CD33^+^ CD3^−^ cells. CD33^+^ CD3^−^ lamina propria myeloid cells (LPMO) [15] were further distinguished from mast cells by lack of or low CD117 expression. Flow cytometric analysis was performed using an LSR II (BD Biosciences) and FACSDiva v6 software (BD Bioscience). Doublets were identified by plotting FSC-A versus FSC-H and excluded from the analysis. As negative controls the (auto)fluorescence of the myeloid cell populations (stained with CD33PE-Cy7/CD3 V450, and CD117 APC (LPMO), respectively) was determined.

### Laser-Capture Microdissection (LMD)

12 µm thick cryosections were prepared from flash frozen colonic tissue samples collected prior to culturing as well as after loss of the epithelial layer (LEL-M 5 h). After transfer to NF 1.0 PEN membrane coated slides (Carl Zeiss MicroImaging, Munich, Germany) sections were treated with DTT (1 mg/ml) for 5 min and stored in 100% EtOH at −20°C. Immediately before laser-capture microdissection, sections were stained with Cresyl Violet (0.1% in 100% EtOH; Sigma) and subsequently dehydrated by immersion in 100% EtOH and Xylene (Roth, Karlsruhe, Germany). LMD was performed for 1 h each using the PALM MicroBeam (Carl Zeiss MicroImaging). Microdissected tissue was collected using opaque AdhesiveCaps (Carl Zeiss MicroImaging).

### Microarray Analysis

RNA was isolated with the RNeasy Micro Kit (Qiagen, Hilden, Germany) according to the manufacturer’s instructions for microdissected samples, using glycogen (Sigma) as a carrier. RNA quantity and integrity was determined using the Agilent 2100 Bioanalyzer (Agilent Technologies, Waldbronn, Germany). All samples had a RNA integrity number (RIN) between 3.1 and 6.6. The mean RIN of RNA samples directly sampled by LMD (t = 0 h) was 5.1±1.67 compared to 3.9±0.75 after 5 h (t = 5 h) incubation. Samples were stored at −80°C until microarray analysis.

Microarray analysis was performed using the whole genome cDNA-mediated Annealing, Selection, extension and Ligation (WG-DASL) Assay (Illumina, San Diego, CA, USA) employing a minimum of 200 ng of total RNA per sample (≥40 ng/µl; input 5 µl). Four replicates were measured for each time point. The analysis was conducted at the Genomics and Proteomics Core facility of the German Cancer Research Center (DKFZ), Heidelberg, Germany. Complete microarray data have been submitted to the GEO repository and are available under the accession number GSE56448.

### Analysis of Microarray Data

Microarray data were normalized by variance stabilizing normalization (vsn) [16]. Differential expression has been calculated by the test implemented in the limma package [17], and P values adjusted for multiple testing by the method of Benjamini and Hochberg [18]. Over-representation analysis of GeneOntology (GO) terms ('biological process' branch only) was calculated by GOstats [19] using the hypergeometric test conditional on the GO structure. Heatmaps were calculated on gene-wise z-transformed data by hierarchical clustering (complete linkage method, Euclidean distance) on rows. All calculations were performed in R v.3.0.0 [20] with the following extension packages: lumi v.2.12.0 [21], limma v.3.16.8 [17], GOstats v.2.26.0 [19], and GO.db v.2.9.0. For comparison with published data [22], we took the list of limma results for all probes from the microarray and selected those genes that had a corrected P value of less than 0.01 and absolute log2-fold change of at least one. This resulted in 439 up-regulated and 297 down-regulated genes, respectively. To calculate significance of overlap, 1000 random lists of length 439 were drawn from all microarray probes in [22] and overlap calculated with genes found to be differentially expressed in the LEL model. The distribution of sizes of intersection sets was used to calculate empirical P values (given as “<0.001” if the observed size was never exceeded by data from any random list).

### TUNEL Assay

Apoptosis was analysed using the TACS 2 TdT-DAB *In Situ* Apoptosis Detection Kit (Trevigen, Gaithersburg, MD, USA). The assay was performed according to the manufacturer’s instructions.

### Ethics Statement

All human studies were approved by the ethics committee of the University of Heidelberg and performed in accordance with the principles laid down in the Declaration of Helsinki. Written informed consent was obtained from the patients.

## Results

### Induction of Inflammatory Gene Expression in Resident Lamina Propria Cells following EDTA-mediated Loss of the Epithelial Layer

**Figure 1:**
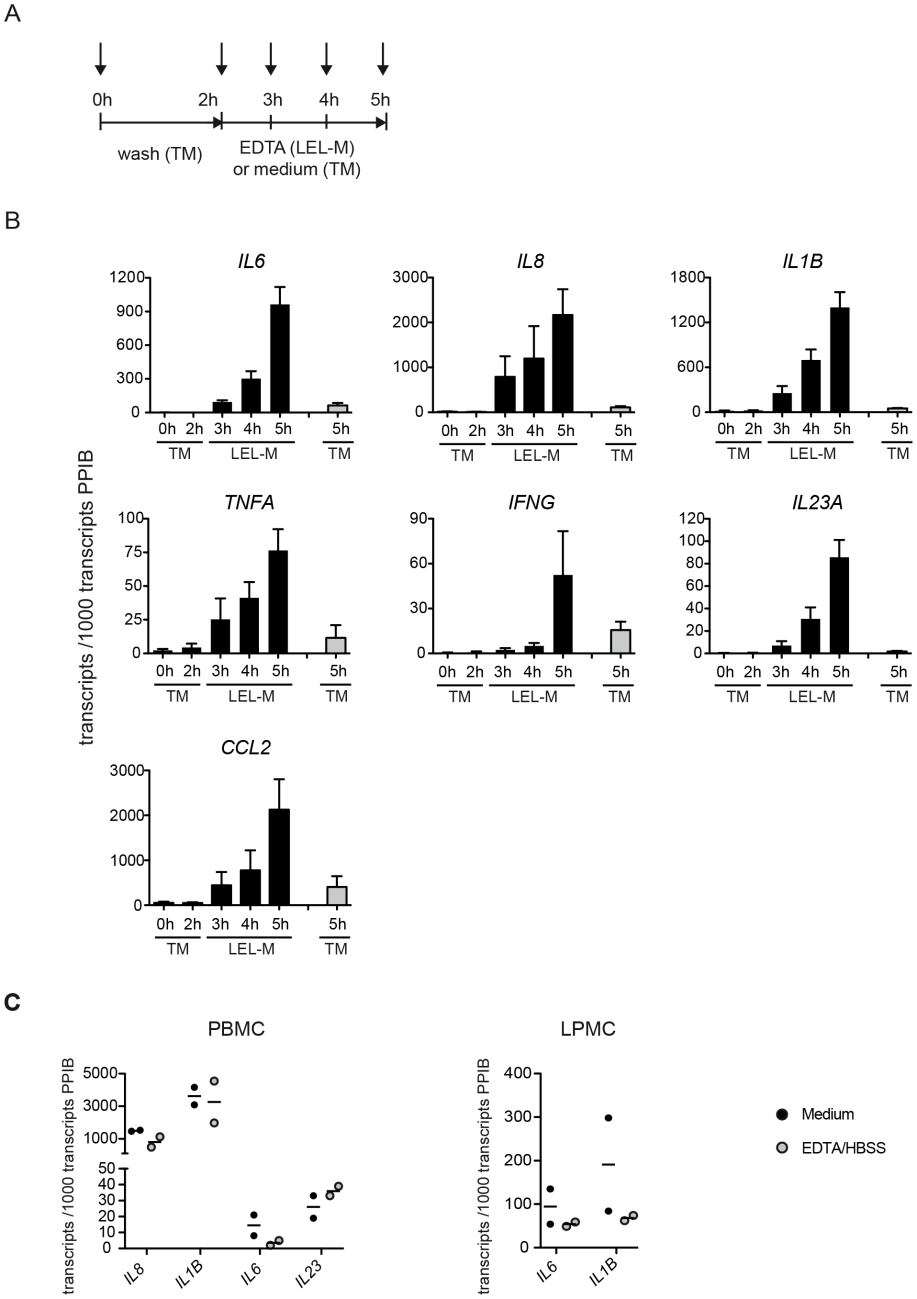
Induction of inflammatory gene expression in resident lamina propria cells following loss of the epithelial layer. (**A**) Time scheme of the LEL model. Arrows indicate time points of tissue collection. Tissue samples were collected prior to culturing (TM t = 0 h), after washing (TM t = 2 h), as well during and after completion of epithelial cell release by EDTA treatment (LEL-M t = 3/4/5 h). As a control, tissue samples were collected from TM cultured for 5 h (TM t = 5 h). (**B**) LEL induces gene expression of *IL1B*, *IL6*, *IL8*, *IL23A*, *TNFA, IFNG*, and *CCL2* in resident lamina propria cells. Transcript levels of cytokines/chemokines were determined by qRT-PCR in tissue samples collected as described in (A). Shown are the mean normalized transcript numbers ± SEM of at least 4 independent experiments. Gray bars represent transcript levels of TM (t = 0/2/5 h), black bars represent transcript levels of LEL-M (t = 3/4/5h). (**C**) EDTA treatment does not induce inflammatory cytokines in PBMC or LPMC. PBMC and LPMC, respectively, were exposed to 0.7 mM EDTA/HBSS or medium (RPMI/2% FCS) for 3 h. Subsequently, transcript levels of *IL6*, *IL8*, *IL1B*, and *IL23A* were determined by qRT-PCR (*IL8* and *IL23A* not tested for LPMC). The results of two independent experiments are shown.

Healthy colonic mucosa of defined size was prepared from freshly obtained human surgical specimens and depleted of epithelial cells by exposure to EDTA under standardized conditions. Mucosal tissue was harvested prior to culturing (total mucosa (TM) t = 0 h), during or immediately after loss of the epithelial layer (“loss of the epithelial layer” mucosa (LEL-M) t = 3/4/5 h); Fig. 1A). As a control, total mucosa (including the epithelial layer) was cultured for 5 h prior to harvesting (TM t = 5 h).

We chose to employ qRT-PCR in order to evaluate the tissue expression of a selected number of “conventional” inflammatory genes. As shown in Fig. 1B, expression of the cytokine genes *IL6, IL8, IL1B, IL23A, TNFA, IFNG* as well as the chemokine gene *CCL2* was markedly upregulated in LEL-M (t = 3/4/5 h) when compared to mucosa prior to culturing, which contained only low numbers of these transcripts. Transcript levels of all inflammatory mediators analysed were also higher in LEL-M at t = 5 h when compared to total mucosa cultured for the same period of time (TM t = 5 h). *IL12B* mRNA was not consistently detected in EDTA treated mucosa (data not shown). Note that treatment of peripheral blood (PBMC) and lamina propria (LPMC) mononuclear cells (isolated by enzymatic digestion), respectively, with EDTA using similar conditions as employed for epithelial cell detachment from mucosal tissue did not cause upregulation of *IL6, IL8, IL1B* and *IL23A* transcript numbers (*CCL2, IFNG, TNFA* not tested) (Fig. 1C).

### Induction of Co-stimulatory Molecules and Pattern Recognition Receptors in Resident Lamina Propria Cells Following EDTA-mediated Loss of the Epithelial Layer

**Figure 2:**
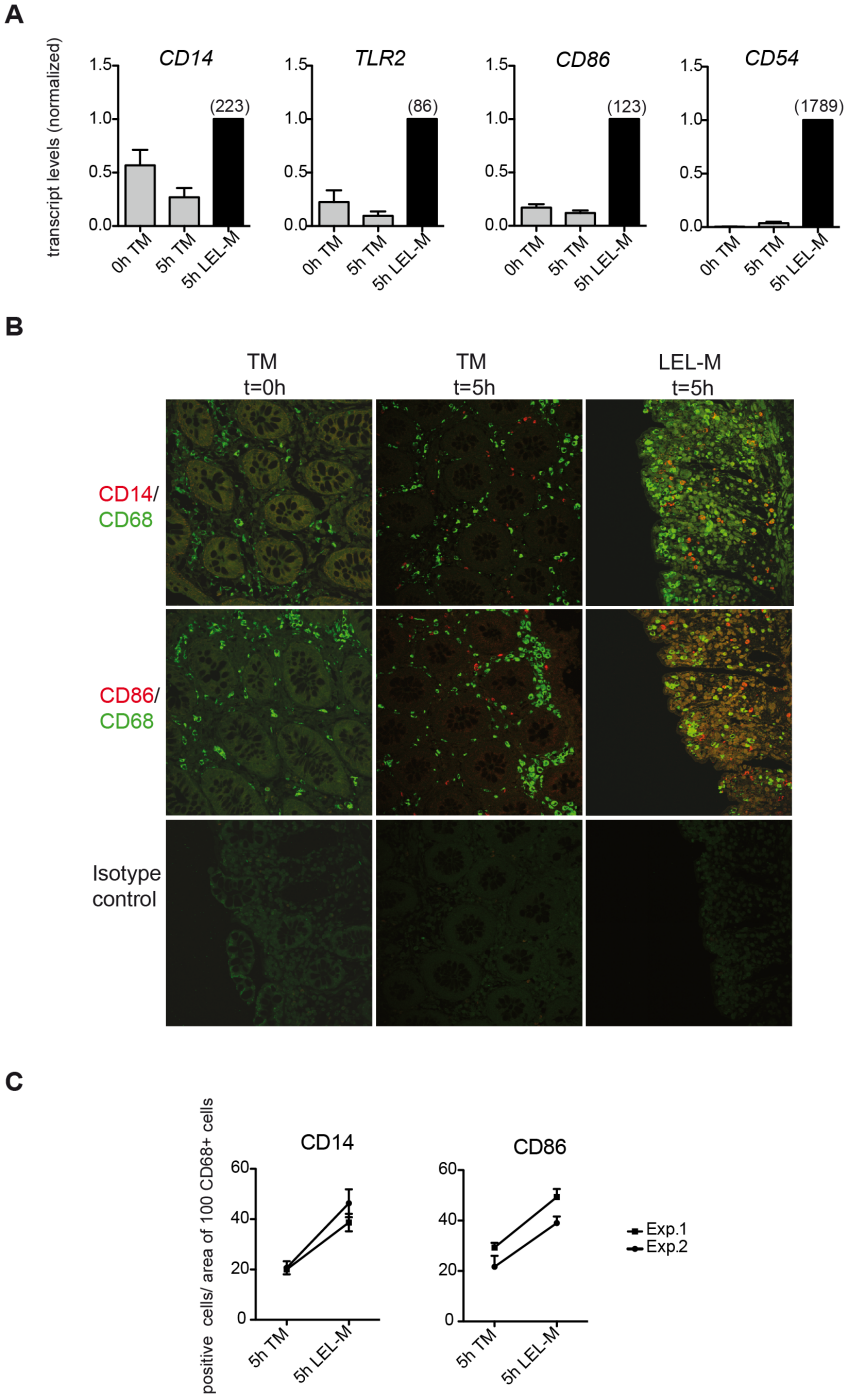
Expression of surface receptors in resident lamina propria cells following loss of the epithelial layer. (**A**) LEL induces gene expression of *CD14*, *CD86*, *TLR2*, and *CD54* in resident lamina propria cells *in situ*. Tissue samples of total mucosa (TM) were collected prior to culturing (0 h TM) and simultaneously from both TM and LEL mucosa after completion of epithelial cell release by EDTA treatment (5 h TM, 5 h LEL-M) (see Fig. 1a). Subsequently, transcript levels of *CD14*, *TLR2*, *CD54*, and *CD86* were determined by qRT-PCR. Transcript numbers (normalized to 1000 transcripts *PPIB*) of “5 h LEL-M” were set to 1, and those of all other conditions were calculated as a fraction/multiple of 1. Shown are the mean normalized transcript numbers ± SEM of three or four independent experiments. Numbers in brackets indicate the absolute transcript numbers normalized to 1000 transcripts *PPIB*. Gray bars represent transcript levels of TM (0 h, 5 h), black bars represent transcript levels of LEL-M (5 h). (**B**) LEL induces protein expression of CD14 and CD86 in resident lamina propria cells *in situ*. Double immunofluorescence staining of CD68 (green) and CD14 or CD86 (red) in healthy total mucosa prior to culturing (TM t = 0 h) and after 5 h of culture (TM t = 5 h) as well as in mucosa immediately after LEL (LEL-M t = 5 h). Colocalization of both antigens is shown by yellow signals in the overlay. Magnification: x40. LEL enhances the expression of CD14 and CD86 on some CD68^+^ and a large pool of CD68^−^ cells. The results represent one of three independent experiments. (**C**) Quantification of CD14^+^ and CD86^+^ cells in TM 5 h and LEL-M 5 h, respectively. The mean numbers (± SEM) of CD14^+^ and CD86^+^ cells, respectively, per area of 100 CD68^+^ cells were determined with three areas/experimental condition being evaluated. Shown are the results of two independent experiments.

Pattern recognition receptors and co-stimulatory molecules are critically involved in innate and adaptive immune responses [23], [24]. Under homeostatic conditions, their expression has been described to be detectable only at low levels in the intestinal lamina propria [10]–[12]. We therefore examined the effect of EDTA-mediated loss of the epithelial layer on the expression of the genes encoding the PRR *CD14*, *TLR2*, as well as the co-stimulatory molecules *CD86* and *CD54* in mucosa cells. As shown in Fig. 2A, transcript levels of these genes were upregulated in mucosal tissue within 3 h after starting to inflict LEL (5 h LEL-M versus 0 h TM) while being not or marginally altered in total mucosa cultured for the same period of time (5 h TM versus 0 h TM).

In parallel, protein expression of CD14 and CD86, respectively, was determined in formalin-fixed tissue samples collected prior to culturing and immediately after epithelial cell depletion by immunohistological analysis. As shown in Fig. 2B, expression of both surface receptors was not detectable in lamina propria cells in the presence of an intact epithelial layer (TM t = 0 h) - in accordance with a previous study [25]. After 5 h of culture of total mucosa (TM t = 5 h) some CD14^+^ and CD86^+^ cells, respectively, which were negative for the macrophage marker CD68 (green fluorescence) appeared in the lamina propria. Note that at this time point signs of epithelial layer disintegration were already detectable by Hematoxylin-Eosin staining (see Figure S1). Importantly, following detachment of epithelial cells (LEL-M t = 5 h) higher numbers of CD14^+^ as well as CD86^+^ CD68^−^ cells than in the medium control (TM t = 5 h) could be observed in the lamina propria (Fig. 2B and 2C). Notably, the expression pattern of CD86 and CD14 in mucosal cells as observed in the organ culture model resembles that in intestinal inflammation *in vivo* (ulcerative colitis) (see Figure S2).

In line with the *in situ* results, expression of CD14 and CD86 was detectable on lamina propria myeloid cells (LPMO; CD33^+^ CD3^−^ CD117^−^) rapidly isolated after epithelial cell detachment (by enzymatic digestion of the lamina propria) as determined by flow cytometry. Expression levels on LPMO were comparable to those on autologous peripheral blood monocytes (PBMO; CD33^+^ CD3^−^) (see Figure S3A). Both myeloid cell populations expressed HLA-DR with significantly higher levels being detectable on LPMO than on PBMO. Note that neither EDTA nor enzymatic treatment (as employed for the isolation of LPL) of PBL resulted in an upregulation of CD14 and CD86 surface expression on PBMO (see Figure S3B, C).

### Early Inflammatory Gene Expression Profile of Resident Lamina Propria Cells

**Figure 3:**
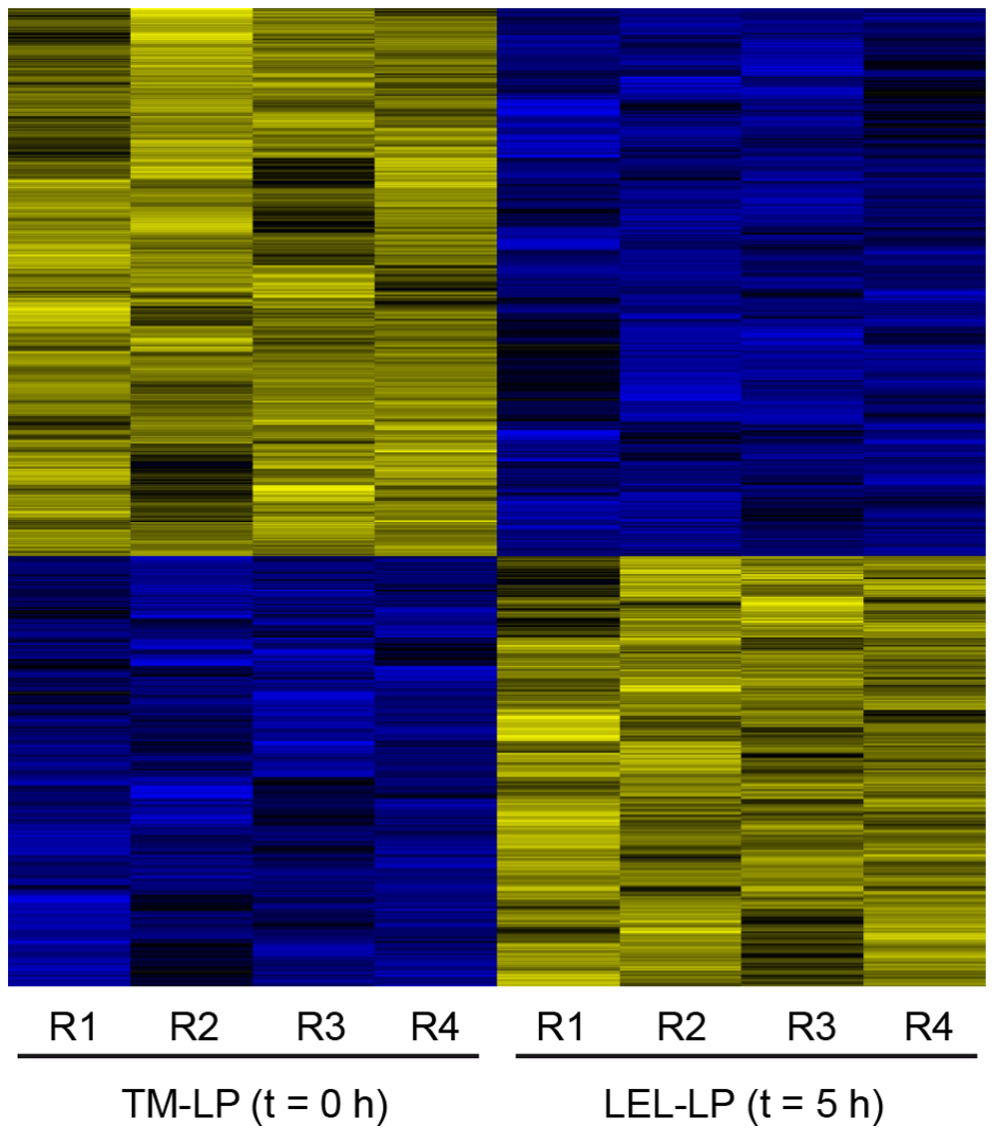
Early inflammatory gene expression profile of resident lamina propria cells. Frozen tissue samples collected prior to culturing (TM 0 h) and immediately after release of the epithelial layer (LEL-M 5 h) were subjected to laser-capture microdissection of the lamina propria. Following RNA extraction, global gene expression profiles of lamina propria cells were obtained by microarray analysis. Shown is a heatmap of significantly regulated genes in LEL-LP 5 h vs. TM-LP 0 h (four matched replicates (R1–R4) for each time point replicates representing four independent experiments). Colors show expression relative to the average (black), higher (yellow), or lower (blue) relative expression, respectively. Data have been transformed to gene-wise zero mean and unit variance (z transform). Rows have been re-ordered by hierarchical clustering (complete linkage, Euclidean distance metrics).

Given that our investigation so far contained the bias of arbitrary selection of inflammatory “markers”, we in addition decided to perform a global gene expression analysis. In order to focus on the features of immunocompetent cells in the intestine with regard to their phenotypic and functional changes over time, frozen tissue samples collected prior to culturing (TM t = 0 h) as well as immediately after EDTA treatment (LEL-M t = 5 h) were subjected to laser-capture microdissection (LMD) of the lamina propria (LP). Subsequently, mRNA was extracted for global gene expression analysis followed by bioinformatic evaluation. As shown in Fig. 3, microarray analysis of four individual experiments (replicates R1–R4 for each of the two experimental conditions (TM-LP t = 0 h and LEL-LP t = 5 h)) yielded reproducible results. Compared to microdissected lamina propria prior to culturing (t = 0 h), 1119 genes were differentially regulated in the lamina propria following EDTA treatment (t = 5 h) with 488 genes being significantly up-, and 631 genes significantly downregulated (cut-off: adjusted p value <0.01). Table 1 lists the top 40 upregulated genes, respectively, ranked by fold change (for a complete list of all differentially regulated genes see Table S1). Importantly, among these significantly upregulated genes were also some of those observed to be upregulated using qRT-PCR (see Fig. 1), namely *IL6*, *IL8*, *IL1B*, *IL23A* and *CCL2*. An increase in gene expression of *IFNG* as well as *TNFA* was also observed though not at a statistically significant level (data not shown). Given that a number of epithelial cell specific genes were included within the dataset of downregulated genes (likely due to some epithelial cell contamination of the microdissected lamina propria preparation), the subsequent functional analysis was at this point restricted to the dataset of significantly upregulated genes only.

**Table 1:** Top 40 upregulated genes in the LEL model.

Gene symbol	logFC^1^	adj. P.Val^2^
*IL6*	5,1	7,50E-05
*CSF3*	5,0	1,84E-05
*C2CD4B*	4,7	5,43E-05
*SERPINA3*	4,5	9,16E-05
*RND1*	4,4	4,81E-06
*FAM71A*	4,3	1,53E-04
*CSF3*	4,2	1,39E-03
*EGR3*	4,2	2,41E-06
*IL8*	4,1	2,69E-04
*C2CD4B*	4,1	1,02E-04
*CXCL2*	4,0	6,15E-06
*ICAM4*	4,0	1,99E-05
*MAFF*	4,0	1,95E-04
*MAFA*	4,0	2,89E-04
*ADAMTS4*	3,9	4,76E-04
*ATF3*	3,9	3,03E-04
*ICAM4*	3,8	2,39E-05
*FOSL1*	3,8	4,85E-06
*ABL2*	3,8	2,14E-04
*ARC*	3,7	6,61E-04
*GEM*	3,7	3,79E-04
*TNFSF9*	3,7	1,64E-05
*AMPD3*	3,7	2,16E-03
*GFPT2*	3,7	5,27E-06
*NR4A1*	3,7	6,55E-03
*MOB4*	3,7	2,09E-03
*CCL21*	3,6	1,39E-03
*UBC*	3,6	1,64E-03
*C2CD4B*	3,6	4,81E-06
*EGR3*	3,6	2,39E-04
*SOCS3*	3,6	8,86E-07
*HAS1*	3,6	2,37E-03
*NA*	3,5	4,99E-04
*RGS16*	3,4	6,37E-05
*NR4A1*	3,4	7,89E-05
*DES*	3,4	4,77E-04
*CALCA*	3,3	3,75E-03
*ATF3*	3,3	4,84E-04
*RRAD*	3,3	2,87E-06
*NFKBIZ*	3,3	2,59E-03

1 log base 2 of fold change.

2 P value from moderated t test, adjusted by the Benjamini & Hochberg method [18].

In order to gain insight into biological processes occurring during the initiation of an inflammatory response in the organ culture model, an analysis of overrepresentation of gene ontology (GO) categories in our dataset of upregulated genes was performed (Table 2: top 30 significantly over-represented GO terms; for a complete list of all significantly over-represented GO terms see Table S2). Among the GO categories identified were many representing basic features of an inflammatory response, namely “regulation of response to stress” [26], “positive regulation of defense response” [27], “regulation of an inflammatory response”, “blood vessel development” [28], “(chemo)taxis” [26] and “interleukin-1 production” [29]. Furthermore, according to this analysis, NF-κB activation, cAMP and cytokine induced responses as well as the unfolded protein response seem to be important signaling events determining initial activation processes in lamina propria cells. In addition, several GO terms identified cover aspects of the innate response to bacteria suggesting that such microorganisms (or molecules derived from the latter) may contribute to the induction of the inflammatory response in the organ culture model. Alternatively, non-bacterial stimuli eliciting expression of similar gene sets may participate in this response. Of note, significant induction of apoptotic processes in lamina propria cells following LEL as proposed by the GO term analysis could not be substantiated experimentally by TUNEL assay (see Figure S4).

**Table 2:** Over-representation of GO terms in dataset of upregulated genes in the LEL model.

GO Term	P value^1^	Count^2^	Size^3^
positive regulation of cell death	1,08E-09	38	442
positive regulation of NF-kappaB transcription factor activity	5,08E-09	17	104
cellular response to biotic stimulus	1,06E-08	17	109
positive regulation of phosphate metabolic process	4,66E-08	45	662
response to bacterium	1,09E-07	29	337
response to nitrogen compound	2,11E-07	42	628
blood vessel development	4,37E-07	34	469
positive regulation of apoptotic process	4,92E-07	29	367
cellular response to hypoxia	8,40E-07	13	86
regulation of sequence-specific DNA binding transcription factor activity	1,10E-06	21	224
response to decreased oxygen levels	1,88E-06	21	226
response to cytokine stimulus	1,96E-06	34	497
cellular response to oxygen levels	2,09E-06	13	93
Hemopoiesis	3,61E-06	34	511
response to unfolded protein	4,21E-06	9	46
Aging	5,81E-06	18	186
regulation of transferase activity	5,99E-06	32	486
Ossification	6,77E-06	23	285
immune system development	7,93E-06	35	557
regulation of phosphorylation	1,14E-05	38	655
negative regulation of multicellular organismal process	1,15E-05	26	358
regulation of response to stress	1,19E-05	30	457
positive regulation of kinase activity	1,69E-05	29	433
negative regulation of apoptotic process	1,72E-05	36	598
Taxis	1,85E-05	36	600
cellular response to lipopolysaccharide	2,60E-05	9	57
interleukin-1 production	3,35E-05	8	45
regulation of inflammatory response	3,36E-05	17	192
toll-like receptor 3 signaling pathway	3,79E-05	10	73
negative regulation of phosphorylation	3,94E-05	18	214

1 P value of hypergeometric test for over-representation (conditional on GO structure).

2 Count: number of GO term associated genes in the dataset of upregulated genes.

3 Size: total number of GO term associated genes.

### Inflammatory Events in the LEL Model Partially Reflect those Occurring in Intestinal Inflammation *in vivo*

To address (1) the *in vivo* relevance of the organ culture model and (2) the central point whether early activation processes occurring in the *in vitro* system differ from events that exist in symptomatic human colonic diseases, our data collected from the *in vitro* systems were subjected to a comparative analysis with published data from microarray analyses from inflamed colonic tissue (ulcerative colitis (UC)). In the absence of any published gene expression profiling studies using microdissected lamina propria cells, UC data sets generated based on the examination of total biopsy tissue were employed for this comparative analysis [22].

**Figure 4:**
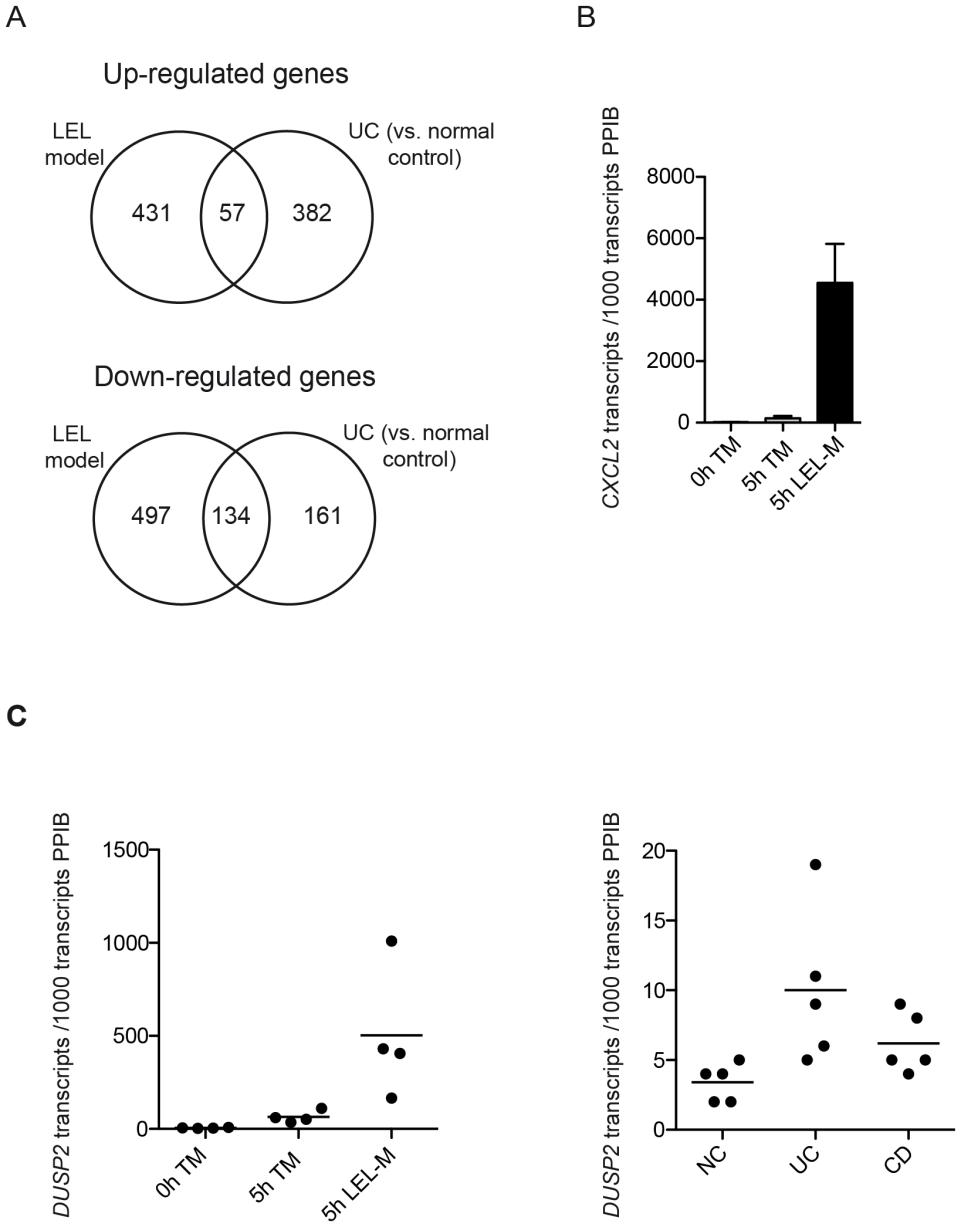
Inflammatory events in the LEL model partially reflect those occurring in intestinal inflammation *in vivo.* (**A**) The overlap of differentially expressed genes in the LEL model *in vitro* and in inflamed tissue obtained from ulcerative colitis patients (vs. normal control tissue) as published in Granlund *et al.* [22] is depicted by a Venn diagram. (**B**) Upregulation of the IBD biomarker gene *CXCL2* in the LEL model. Tissue samples of total mucosa (TM) were collected prior to culturing (0 h TM). Furthermore, tissue samples were collected simultaneously from both TM and LEL mucosa after completion of epithelial cell release by EDTA treatment (5 h TM, 5 h LEL-M) (see Fig. 1a). Subsequently, transcript levels of *CXCL2* were determined by qRT-PCR. Shown are the mean normalized transcript numbers ± SEM of three to four independent experiments. Gray bars represent transcript levels of TM (0 h, 5 h), the black bar represents transcript levels of LEL-M (5 h). (**C**) Identification of *DUSP2* as a novel gene upregulated in intestinal inflammation. *Left panel:* Tissue samples of total mucosa (TM) were collected prior to culturing (0 h TM). Furthermore, tissue samples were collected simultaneously from both TM and LEL mucosa after completion of epithelial cell release by EDTA treatment (5 h TM, 5 h LEL-M) (see Fig.1a). Subsequently, transcript levels of *DUSP2* were determined by qRT-PCR. Shown are normalised transcript numbers (including mean) as determined in four independent experiments. *Right panel:* Transcript levels of *DUSP2* were determined in transmural tissue samples of normal (NC) as well as of inflamed (UC, CD) gut by qRT-PCR. Shown are normalized transcript numbers (including mean) as determined in five different tissue samples of NC, UC, and CD, respectively.

As illustrated in the Venn diagram (Fig. 4A), 57 of 488 significantly upregulated and 134 of 631 downregulated genes in our *in vitro* model were also found to be upregulated and downregulated, respectively, in active UC samples when compared to normal (non-inflamed) gut samples [22]. This corresponds to a significant enrichment of genes differentially expressed in UC (as identified by Granlund *et al.* [22]) in our dataset (p<0.001 for up- and downregulated genes, respectively, as determined by a permutation test with 1000 lists of random genes). Among the shared upregulated genes (see Table S3) were also biomarkers for clinical and/or endoscopic disease activity in UC, such as *IL8*, *CXCL2*, *LCN2*, and *MMP9* [30]–[32]. In addition to *IL8* (see Fig. 1), increased *CXCL2* gene expression in LEL-M (t = 5 h) vs. TM (t = 0 h and t = 5 h) was confirmed by qRT-PCR (Fig. 4B; other markers not tested). As expected, *S100A8* and *S100A9* genes, IBD biomarkers preferentially expressed in peripheral blood myeloid cells (granulocytes, monocytes) [33] migrating into the tissue in response to inflammatory stimuli, were not observed to be upregulated in the LEL model. Notably, a comparison of our dataset with publicly available results (Top 200 differentially regulated genes) of a meta-analysis of IBD gene expression profiles [34] gave similar results (data not shown). These observations clearly demonstrate that key inflammatory events as observed in intestinal inflammation *in vivo* are at least partially reproduced by the organ culture model *in vitro*.

Not surprisingly, the majority of differentially regulated genes in the organ culture model differ from those differentially regulated in active UC (Fig. 4A) [22]. Their potential clinical relevance – in particular with respect to the initial phase of an intestinal inflammatory response- needs to be further evaluated. In this regard it is interesting to note that several of the genes detected to be solely upregulated in our organ culture model have been prioritized as key genes linked to gene loci identified to be associated with UC (or inflammatory bowel disease (IBD) in general) in genome wide association studies [35] (GWAS; see Table S4).

Furthermore, for one of the genes observed to be significantly upregulated in the LEL model, dual specific phosphatase 2 (*DUSP2*), we tried to confirm its differential expression in intestinal inflammation *in vivo*. DUSP2, a regulator of MAP kinases, is induced in human leucocytes following stimulation. It may potentially represent an important signaling module involved in the initiation of intestinal inflammation as it has been demonstrated to promote inflammatory responses in rheumatoid arthritis in mice [36], [37]. As shown in Fig. 4C, induction of *DUSP2* expression in the LEL model as observed by global gene expression profiling was confirmed by qRT-PCR (left panel). Importantly, in agreement with this result in the LEL model *in vitro*, significantly higher expression levels of this gene were observed in inflamed tissue of UC vs. normal control tissue (NC) *in vivo* (p<0.05). Increased *DUSP2* transcript numbers were also detected in inflamed CD tissue, however at a lower level. Note that the low induction of transcript levels of *DUSP2* in UC and CD vs. NC tissue when compared to the LEL model may be due to the fact that different types of tissue were used for these analyses (UC/CD/NC: transmural tissue; LEL model: mucosa).

## Discussion

Inflammation represents a fundamental defense response of the body to infection and tissue damage, which aims at the restoration of homeostasis [26]. In the human intestinal mucosa, molecular processes determining the onset of an inflammatory response in resident lamina propria cells are as yet largely unknown. However, knowledge of these processes would not only help to understand basic principles of the initiation of an intestinal immune response but also potentially promote the identification of novel drug targets for remission maintaining therapy and/or the discovery of biomarkers predicting relapse in IBD [38]. Importantly, it could also provide the basis for unraveling etiologically relevant alterations of early inflammation in IBD.

Here, we characterized the initiation of an inflammatory response in resident lamina propria cells in a human intestinal organ culture model. In this model, an inflammatory response was initiated in lamina propria cells following EDTA mediated loss of the epithelial layer. Damage of the epithelial layer due to disruption of tight junctions, epithelial cell death, erosions, and ulcers represents an early pathogenic event in intestinal inflammation *in vivo*, e.g. in acute enteric infections caused by invasive or toxin- producing pathogens [39]–[42]. It is associated with inflammatory cellular infiltrates of the mucosal tissue, which are largely missing in infections caused by non-invasive pathogens that do not induce significant epithelial cell damage [43], [44]. Furthermore, aphthoid lesions represent one of the earliest pathological mucosal manifestations in Crohn's disease [45].

The inflammatory response elicited in resident lamina propria cells following loss of the epithelial layer (LEL) was characterized by a rapid upregulation of canonical inflammatory mediators, e.g. *IL1B*, *IL8*, as well as co-stimulatory and pattern recognition receptors. With regard to the latter our results provide at least partial mechanistic insight into the previous observation that lamina propria myeloid cells migrated out of the intestinal mucosa during a 12 h culture period following epithelial cells depletion express these receptors [46]. Information on the global transcriptional response induced in the LEL model was obtained by microarray analysis of laser-capture microdissected lamina propria.

Insight into molecular processes including signaling pathways involved in the initiation of an intestinal inflammatory response in resident lamina propria cells was provided by bioinformatic GO term analysis of the set of differentially regulated genes in this model. While -according to this analysis- bacterial stimuli (likely present in the mucosal culture despite extensive washing steps) may contribute to the induction of this inflammatory response they are most likely not sufficient for triggering the latter given the low/lack of homeostatic expression of pattern recognition receptors and associated signaling molecules in lamina propria cells [10]–[13]. Furthermore, inflammatory gene expression was higher in LEL mucosa when compared to total mucosa, which was exposed to at least the same amount of potentially contaminating bacterial stimuli being present during culture for the same period of time (see Fig. 1B). Further studies using the LEL model may provide insight into upstream stimuli, e.g. danger molecules like ATP, mediating the initiation of an inflammatory response in lamina propria cells. In particular, it will be tested whether loss of epithelial cell derived factors, e.g. IL-10, TGF-β, which have been shown to contribute to the control of intestinal inflammation [47]–[49], is sufficient for activation of the latter cell population.

Many of the signaling events (e.g. NF-κB activation, response to cytokines) identified to contribute to early intestinal inflammatory responses in the LEL model are also known to be involved in later, chronic stages of intestinal inflammation suggesting that their activity could potentially be maintained for a prolonged period of time. This observation is in agreement with findings of a recent study showing that changes in (inflammatory) gene expression in peripheral blood leucocytes following severe injury in humans occur within hours and are maintained for up to one to three months until recovery of the patients [50]. Importantly, gene sets associated with the unfolded protein response (UPR) were found to be overrepresented in the dataset of upregulated genes in the LEL model. This is the first indication that the UPR is not only induced in epithelial cells during human chronic intestinal inflammation - as previously described [51], [52] - but also in LP cells at the onset of an intestinal inflammatory response. Hence, genetic susceptibility for IBD linked to hypomorphic variants of the UPR-associated gene *XBP1* [51] may be associated with dysregulation of the UPR not only in epithelial but also lamina propria cells. Importantly, we have collected evidence that the transcription factor C/EBPβ, a target of the UPR [53], is rapidly upregulated in lamina propria myeloid cells in intestinal inflammation (manuscript in preparation).

The relevance of the LEL model with regard to key inflammatory events occurring in inflammation *in vivo* is underlined by the observation that the set of differentially expressed genes in this model partially overlaps with that in inflamed tissue from UC patients (vs. normal colonic mucosa) as determined by Granlund *et al*. [22]. Importantly, among the genes upregulated in the organ culture model were also biomarkers for clinical or endoscopic disease activity in ulcerative colitis [30]–[32].

Clearly, the majority of genes observed to be differentially expressed in in the organ culture model differed from those differentially expressed in active ulcerative colitis and *vice versa*. This is not unexpected given that (1) the LEL model reflects the initial phase of inflammation whereas UC samples subject to gene expression profiling were taken during symptomatic stages of the disease (2) immigration of blood monocytes and granulocytes into inflamed tissue as taking place *in vivo* [26], [54] cannot occur in the LEL model; (3) UC tissue samples are often obtained from patients receiving anti-inflammatory drug treatment, which may modulate inflammatory gene expression; (4) gene expression profiling of the LEL model was performed employing microdissected lamina propria in order to focus the analysis on immunocompetent cells located in this compartment, while microarray analysis of human intestinal tissue collected from UC patients was conducted with total mucosa; (5) IBD-linked genetic polymorphisms may be associated with a dysregulated inflammatory response in UC patients.

The clinical relevance of the genes differentially regulated solely in the LEL model is supported by the fact that several of these genes have been linked to UC/IBD susceptibility loci as identified by GWAS [35] (see Table S4). While differential expression of most of these genes - though not detected by global gene expression profiling- has been demonstrated in IBD in previous studies, differential expression of one of these IBD loci associated genes, *FOSL1*, in human intestinal inflammatory responses is reported here for the first time. FOSL1 belongs to the AP1 family of transcription factors [55]; its overexpression in mice has been demonstrated to ameliorate DSS-induced colitis [56]. Application of the LEL model to non-inflamed mucosa in UC vs. non-IBD may allow to determine a potentially altered regulation of *FOSL1* expression and other IBD loci associated genes in these patients thereby promoting the establishment of genotype-phenotype correlations.

Notably, *DUSP2*, a gene upregulated in the LEL model but not in IBD gene expression profiling datasets of Granlund *et al.* and the meta-analysis by Clark *et al.* (as far as data are available), was confirmed to be upregulated in intestinal inflammation *in vivo*. This result further supports the *in vivo* relevance of novel inflammation related genes as detected in the LEL model. Given its pro-inflammatory role in a murine model of rheumatoid arthritis, DUSP2 may positively regulate inflammatory responses also in intestinal immune cells [37].

Another implication of our findings relates to the interpretation of results gained in experiments using enzymatically isolated “resting” lamina propria leucocytes. Most protocols employed for the isolation of these cells include EDTA treatment of mucosal specimens for release of epithelial cells [14], [57]–[59] as it has been applied in this study. According to our results cells subjected to this treatment during the isolation procedure may not exist in a resting state as observed under homeostatic conditions *in vivo* but rather exhibit features of activated cells.

In summary, molecular events associated with the initiation of an inflammatory response in human lamina propria cells are characterized for the first time using an intestinal organ culture model. The organ culture model may represent a valuable tool to elucidate molecular mechanisms underlying this initial activation of lamina propria cells in man. Furthermore, when applied to non-inflamed mucosa of IBD patients, it may help to determine potential alterations of the early mucosal inflammatory response in these patients and thereby support the discovery of disease-specific drug targets. Finally, it may be useful for identifying general (non-specific) drug targets for remission-maintaining therapy for these diseases.

## Supporting Information

Figure S1 **Hematoxylin-Eosin staining of total mucosa (TM) and mucosa depleted of epithelial cells (LEL-M).** Signs of epithelial layer disintegration are detectable in TM cultured for 5 h (TM 5 h) in comparison to TM prior to culturing (TM 0 h).
(TIF)

Figure S2 **Double immunofluorescence staining of CD68 (green) and CD14 or CD86 (red) in inflamed tissue in ulcerative colitis.** Co-localization of both antigens is shown by yellow signals in the overlay. Magnification: ×40.
(TIF)

Figure S3 **CD14 and CD86 are expressed on lamina propria myeloid cells (LPMO) rapidly isolated after LEL. (A)** LPMO were rapidly isolated by enzymatic tissue digestion after detachment of the epithelial cell layer (LEL). Subsequently, surface expression of CD14 and CD86 was analyzed on PBMO (upper panel) and LPMO (lower panel) by flow cytometry. *Dot blots:* A gate was set on CD33^+^ CD3^−^ CD117^−^ myeloid cells (blue). *Histograms:* Shown are the expression levels of HLA-DR, CD14 and CD86 on CD33^+^CD3^−^CD117^−^ PBMO and LPMO, respectively. Results are representative of two independent experiments. **(B)** Treatment with EDTA does not affect CD14 and CD86 surface expression on PBMO. PBL were cultured in RPMI/10% FCS/antibiotics, HBSS/antibiotics, or HBSS/EDTA 0.7 mM/antibiotics for 1.5 h. Surface expression of CD14 and CD86 on CD33^+^CD3^−^ PBMO was determined by flow cytometry. Shown is the mean fluorescence intensity (MFI) of one of two independent experiments showing similar results. (**C**) Treatment with Collagenase/DNAse does not affect CD14 and CD86 surface expression on PBMO. PBMC were cultured in RPMI/2% FCS/antibiotics in the absence or presence of collagenase IV (45 U/ml)/DNAse I (27 U/ml) for 1.5 h. Surface expression of CD14 and CD86 on CD33^+^ CD3^−^ PBMO was determined by flow cytometry. Shown are % MFI of the untreated controls (100%) of two independent experiments.
(TIF)

Figure S4 **Apoptosis is not significantly induced in lamina propria cells following LEL.** The occurrence of apoptosis during the LEL organ culture was determined using an *in situ* terminal deoxynucleotidyl transferase dUTP nick end labeling (TUNEL) assay. Images show colonic cryosections at t = 0 h (TM) and t = 5 h (LEL-M). Apoptotic cells containing fragmented DNA (thereby indicating apoptosis) are stained brown with 3,3'-diaminobenzidine. Sections are counterstained with Methyl Green. The positive control was achieved with TACS-Nuclease™. Results are representative of two independent experiments.
(TIF)

Table S1 **Complete list of all differentially regulated genes.**
(XLSX)

Table S2 **Complete list of all significantly over-represented GO terms.**
(XLSX)

Table S3 **List of overlapping genes upregulated in the LEL model (LEL-M 5 h vs. TM 0 h) and in UC vs. normal control according to Granlund** ***et al.*** [22] **.**
(DOCX)

Table S4 **Key genes of IBD associated gene loci as described by Jostins** ***et al.*** [35] **included in the set of upregulated genes in the LEL model but not in UC vs. normal control according to Granlund** ***et al*** **.** [22] **.**
(DOCX)
