# Correction: Initiation of an Inflammatory Response in Resident Intestinal Lamina Propria Cells -Use of a Human Organ Culture Model

**DOI:** 10.1371/journal.pone.0105859

**Published:** 2014-08-11

**Authors:** The PLOS ONE Staff

There are multiple errors in this article:

First, there is an error in the Financial Disclosure (FD) statement of this article. The website link provided for the funding website is incorrect. Please refer to the correct FD statement below:

“This work was funded by the Deutsche Forschungsgemeinschaft (www.dfg.de; SFB 938, project O, M, and Z2). The funders had no role in study design, data collection and analysis, decision to publish, or preparation of the manuscript.”

Second, the last sentence in the “Materials and Methods” section, sub-heading “Loss of Epithelial Layer” (LEL) Organ Culture” is incorrect. The correct sentence should read:

“This incubation was repeated two times with two washing steps (10 min in HBSS/antibiotics) after each incubation period.”

Third, Figure S4, Table S3 and Table S4 are labeled incorrectly. Please refer to the correct labels here:

Figure S4 Apoptosis is not significantly induced in lamina propria cells following LEL. The occurrence of apoptosis during the LEL organ culture was determined using an in situ terminal deoxynucleotidyl transferase dUTP nick end labeling (TUNEL) assay. Images show colonic cryosections at t  =  0 h (TM) and t  =  5 h (LEL-M). Apoptotic cells containing fragmented DNA (thereby indicating apoptosis) are stained brown with 3,3'-diaminobenzidine. Sections are counterstained with Methyl Green. The positive control was achieved with TACS-Nuclease™. Results are representative of two independent experiments.

Table S3 List of overlapping genes upregulated in the LEL model (LEL-M 5 h vs. TM 0 h) and in UC vs. normal control according to Granlund et al.

Table S4 Key genes of IBD associated gene loci as described by Jostins et al. [35] included in the set of upregulated genes in the LEL model but not in UC vs. normal control according to Granlund et al.
