# Supplementary figures and images for: Initiation of an Inflammatory Response in Resident Intestinal Lamina Propria Cells -Use of a Human Organ Culture Model
Source: PLoS One. 2014 May 19;9(5):e97780. doi: 10.1371/journal.pone.0097780 (PMC4026413; doi:10.1371/journal.pone.0097780)

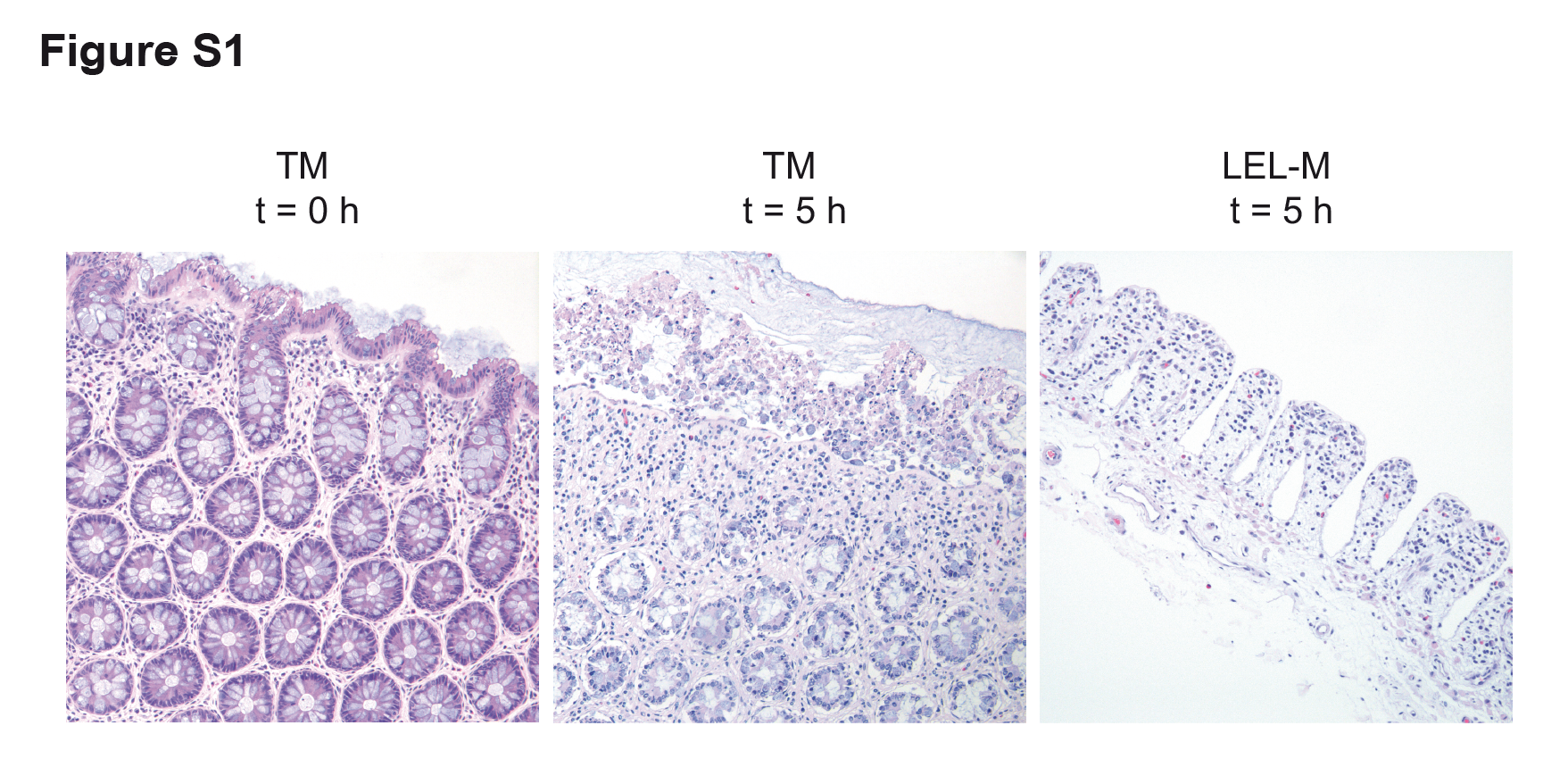

Supplement: Figure S1 — Hematoxylin-Eosin staining of total mucosa (TM) and mucosa depleted of epithelial cells (LEL-M). Signs of epithelial layer disintegration are detectable in TM cultured for 5 h (TM 5 h) in comparison to TM prior to culturing (TM 0 h). (TIF) [file pone.0097780.s001.tif]

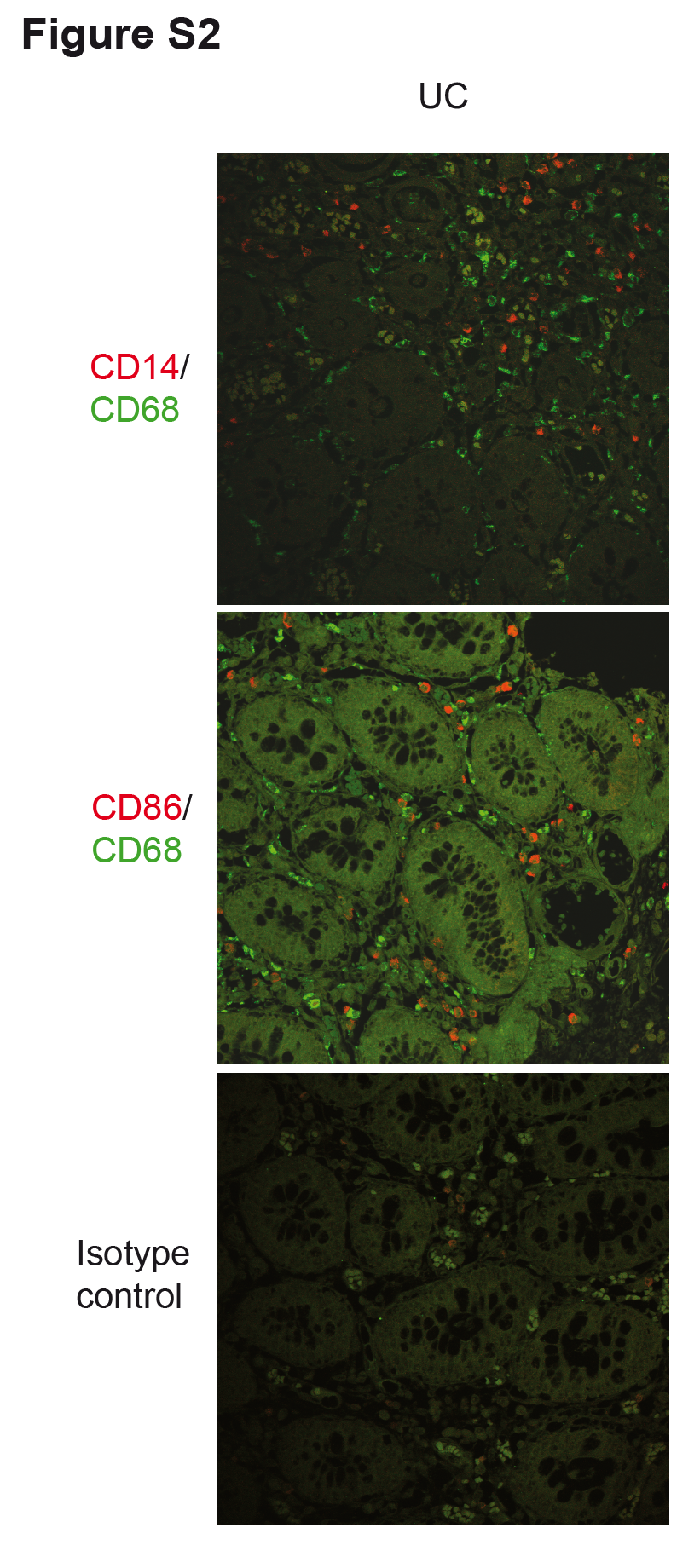

Supplement: Figure S2 — Double immunofluorescence staining of CD68 (green) and CD14 or CD86 (red) in inflamed tissue in ulcerative colitis. Co-localization of both antigens is shown by yellow signals in the overlay. Magnification: ×40. (TIF) [file pone.0097780.s002.tif]

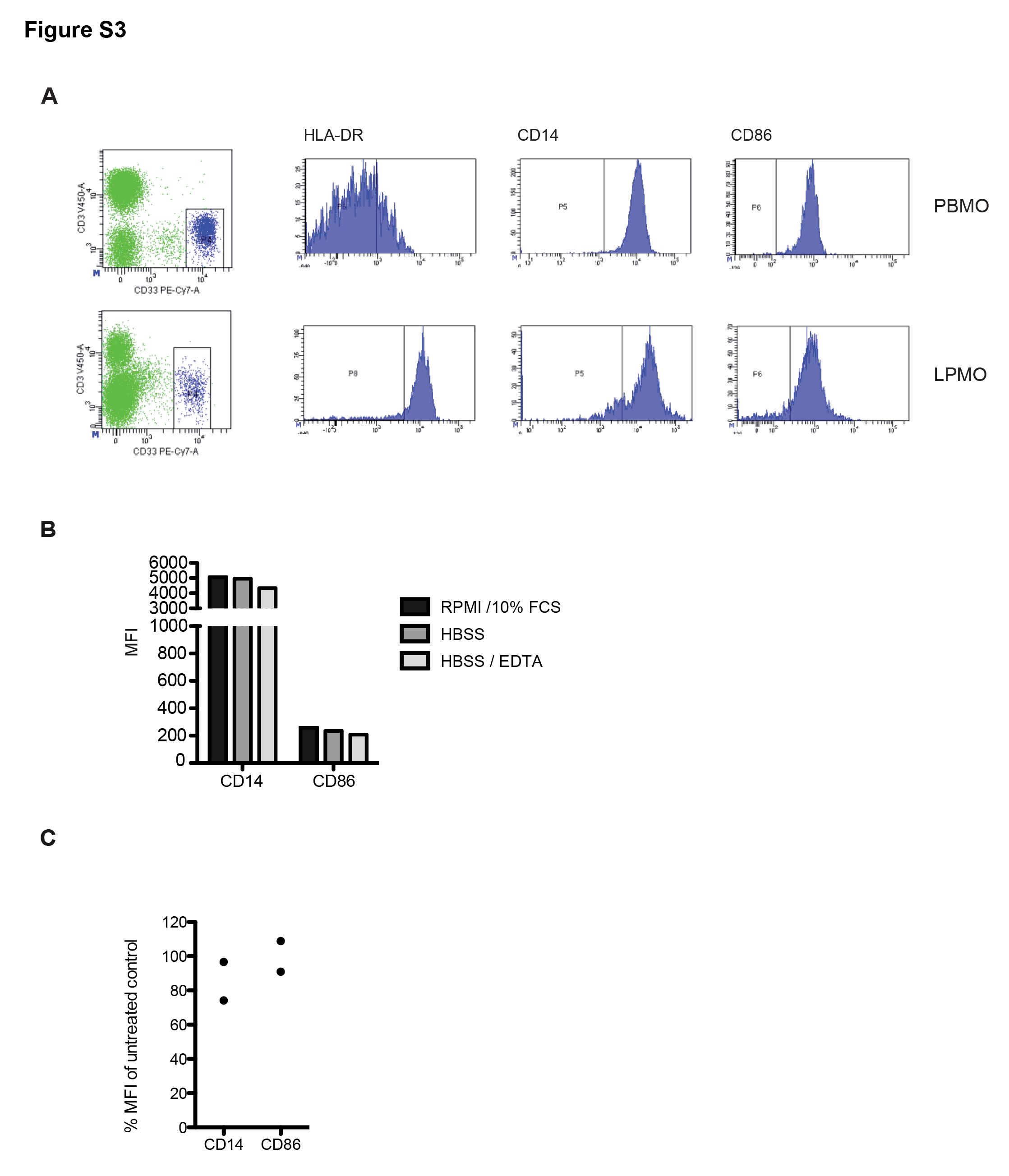

Supplement: Figure S3 — CD14 and CD86 are expressed on lamina propria myeloid cells (LPMO) rapidly isolated after LEL. (A) LPMO were rapidly isolated by enzymatic tissue digestion after detachment of the epithelial cell layer (LEL). Subsequently, surface expression of CD14 and CD86 was analyzed on PBMO (upper panel) and LPMO (lower panel) by flow cytometry. Dot blots: A gate was set on CD33+ CD3− CD117− myeloid cells (blue). Histograms: Shown are the expression levels of HLA-DR, CD14 and CD86 on CD33+CD3−CD117− PBMO and LPMO, respectively. Results are representative of two independent experiments. (B) Treatment with EDTA does not affect CD14 and CD86 surface expression on PBMO. PBL were cultured in RPMI/10% FCS/antibiotics, HBSS/antibiotics, or HBSS/EDTA 0.7 mM/antibiotics for 1.5 h. Surface expression of CD14 and CD86 on CD33+CD3− PBMO was determined by flow cytometry. Shown is the mean fluorescence intensity (MFI) of one of two independent experiments showing similar results. (C) Treatment with Collagenase/DNAse does not affect CD14 and CD86 surface expression on PBMO. PBMC were cultured in RPMI/2% FCS/antibiotics in the absence or presence of collagenase IV (45 U/ml)/DNAse I (27 U/ml) for 1.5 h. Surface expression of CD14 and CD86 on CD33+ CD3− PBMO was determined by flow cytometry. Shown are % MFI of the untreated controls (100%) of two independent experiments. (TIF) [file pone.0097780.s003.tif]

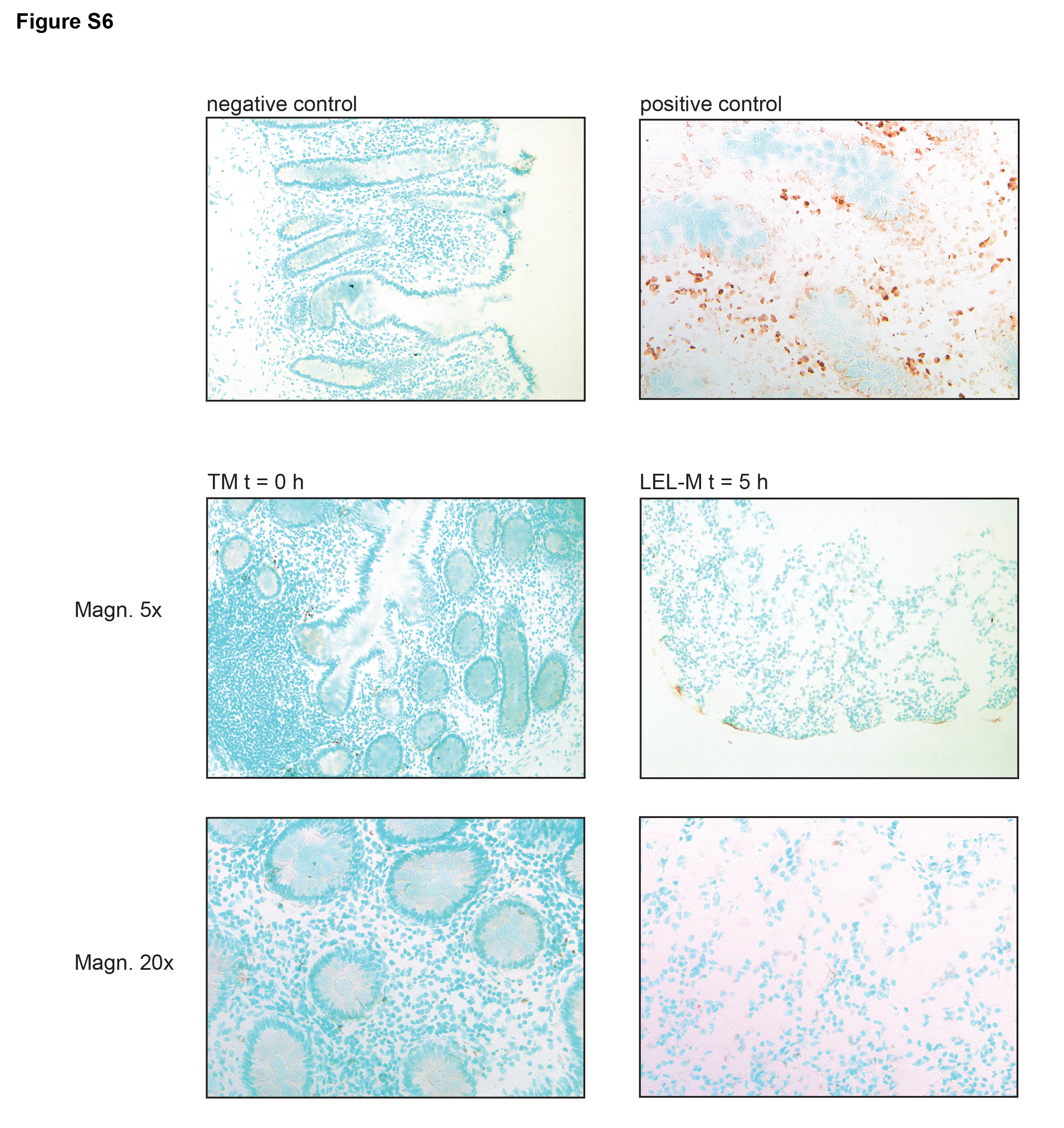

Supplement: Figure S4 — Apoptosis is not significantly induced in lamina propria cells following LEL. The occurrence of apoptosis during the LEL organ culture was determined using an in situ terminal deoxynucleotidyl transferase dUTP nick end labeling (TUNEL) assay. Images show colonic cryosections at t = 0 h (TM) and t = 5 h (LEL-M). Apoptotic cells containing fragmented DNA (thereby indicating apoptosis) are stained brown with 3,3'-diaminobenzidine. Sections are counterstained with Methyl Green. The positive control was achieved with TACS-Nuclease™. Results are representative of two independent experiments. (TIF) [file pone.0097780.s004.tif]
