# Supplementary figures and images for: Correction: Initiation of an Inflammatory Response in Resident Intestinal Lamina Propria Cells -Use of a Human Organ Culture Model
Source: PLoS One. 2014 Aug 11;9(8):e105859. doi: 10.1371/journal.pone.0105859 (PMC4128765; doi:10.1371/journal.pone.0105859)

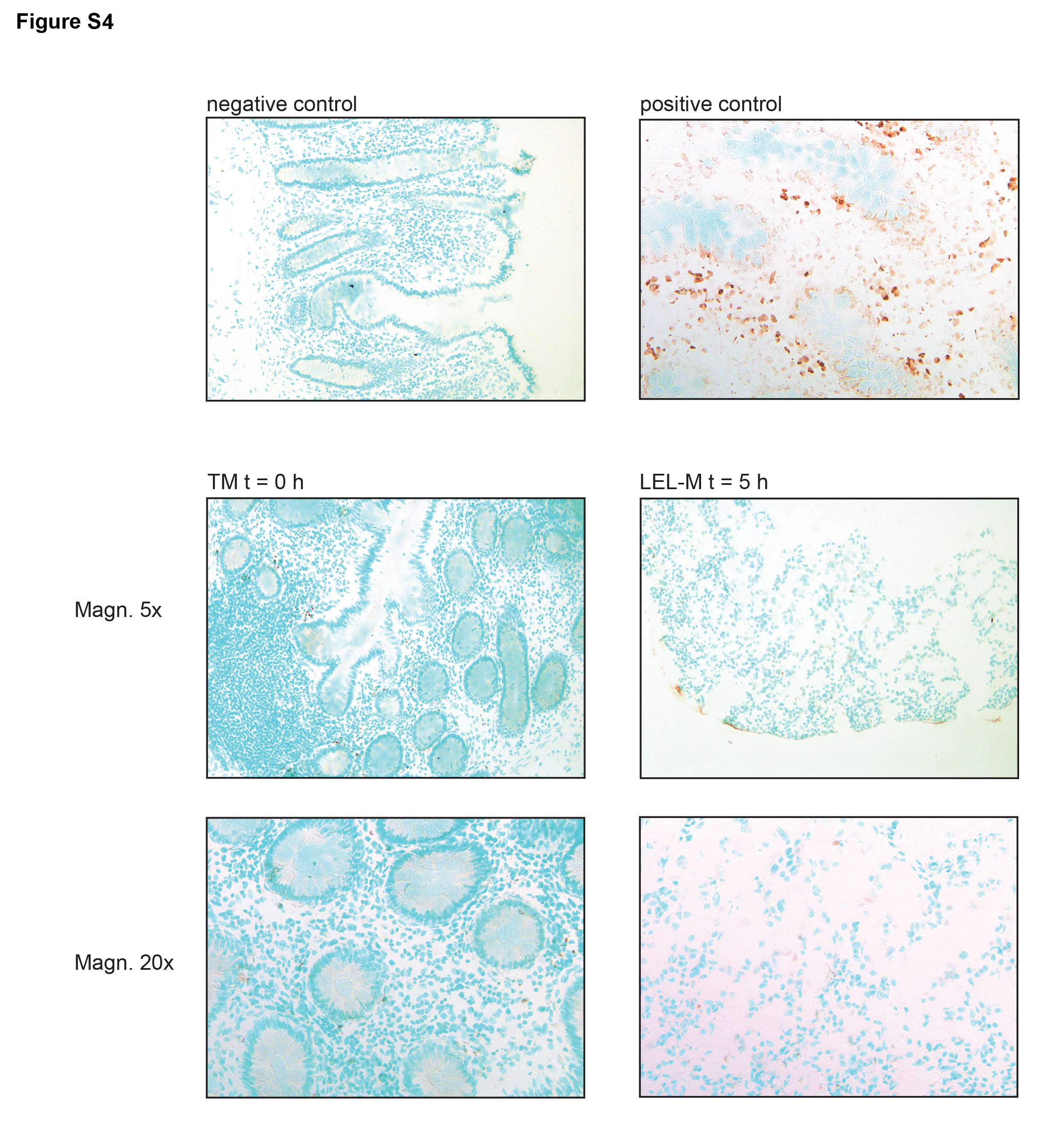

Supplement: Figure S4 — Apoptosis is not significantly induced in lamina propria cells following LEL. The occurrence of apoptosis during the LEL organ culture was determined using an in situ terminal deoxynucleotidyl transferase dUTP nick end labeling (TUNEL) assay. Images show colonic cryosections at t = 0 h (TM) and t = 5 h (LEL-M). Apoptotic cells containing fragmented DNA (thereby indicating apoptosis) are stained brown with 3,3'-diaminobenzidine. Sections are counterstained with Methyl Green. The positive control was achieved with TACS-Nuclease™. Results are representative of two independent experiments. [file pone.0105859.s001.tif]
