# Supplementary material for: Correction: Initiation of an Inflammatory Response in Resident Intestinal Lamina Propria Cells -Use of a Human Organ Culture Model
Source: PLoS One. 2014 Aug 11;9(8):e105859. doi: 10.1371/journal.pone.0105859 (PMC4128765; doi:10.1371/journal.pone.0105859)
Supplement: Table S3 — List of overlapping genes upregulated in the LEL model (LEL-M 5 h vs. TM 0 h) and in UC vs. normal control according to Granlund et al. [file pone.0105859.s002.docx]

**Table S3.** **List of overlapping genes up-regulated in the LEL model (LEL-M 5h vs. TM 0h) and in UC vs. normal control according to Granlund *et al.*[**[**1**](#_ENREF_1)**]**

| \|  \| \| --- \| \| C2CD4B \| \| CXCL2 \| \| CD83 \| \| SERPINE1 \| \| IL1B \| \| CXCL1 \| \| IL6 \| \| NR4A2 \| \| SERPINA3 \| \| CCL2 \| \| PFKFB3 \| \| ZC3H12A \| \| PLAUR \| \| PIM3 \| \| TAGLN \| \| SOD2 \| \| NAMPT \| \| IL8 \| \| ELOVL5 \| \| ATF3 \| \| LYPD5 \| \| PDLIM7 \| \| GEM \| \| PDE4B \| \| EGR1 \| \| SLC2A3 \| \| PHLDA1 \| \| CSRNP1 \| \| FOSB \| \|  \| | \|  \| \| --- \| \| MMP9 \| \| TGM2 \| \| TNFAIP6 \| \| SIK1 \| \| BCL6 \| \| LBH \| \| FLNC \| \| BHLHE40 \| \| NCOA7 \| \| NFKBIZ \| \| ADM \| \| SLC7A5 \| \| IFI16 \| \| PCDH17 \| \| C8orf4 \| \| IER3 \| \| TNFRSF12A \| \| C3 \| \| SRGN \| \| TOX2 \| \| PTGS2 \| \| GREM1 \| \| UBD \| \| IRAK3 \| \| TYMP \| \| BHLHE22 \| \| CCL3 \| \| NPTX2 \| |
| --- | --- | --- | --- | --- | --- | --- | --- | --- | --- | --- | --- | --- | --- | --- | --- | --- | --- | --- | --- | --- | --- | --- | --- | --- | --- | --- | --- | --- | --- | --- | --- | --- | --- | --- | --- | --- | --- | --- | --- | --- | --- | --- | --- | --- | --- | --- | --- | --- | --- | --- | --- | --- | --- | --- | --- | --- | --- | --- | --- | --- | --- |

1. Granlund A, Flatberg A, Ostvik AE, Drozdov I, Gustafsson BI, et al. (2013) Whole genome gene expression meta-analysis of inflammatory bowel disease colon mucosa demonstrates lack of major differences between Crohn's disease and ulcerative colitis. PLoS One 8: e56818.
