# Supplementary material for: Correction: Initiation of an Inflammatory Response in Resident Intestinal Lamina Propria Cells -Use of a Human Organ Culture Model
Source: PLoS One. 2014 Aug 11;9(8):e105859. doi: 10.1371/journal.pone.0105859 (PMC4128765; doi:10.1371/journal.pone.0105859)
Supplement: Table S4 — Key genes of IBD associated gene loci as described by Jostins et al. [35] included in the set of upregulated genes in the LEL model but not in UC vs. normal control according to Granlund et al. [file pone.0105859.s003.docx]

**Table S4:** Key genes of IBD associated gene loci as described by Jostins et al.[[1](#_ENREF_1)] included in the set of up-regulated genes in the LEL model but not in UC vs. normal control according to Granlund et al.[[2](#_ENREF_2)]

| **key genes[**[**1**](#_ENREF_1)**]** | **SNPs* [**[**1**](#_ENREF_1)**]** |
| --- | --- |
|  |  |
| CD40 | rs1569723 |
| CSF2 | rs2188962 |
| FOSL1 | rs2231884 |
| FYN | rs3851228 |
| ICAM1 | rs11879191 |
| NFIL3 | rs4743820 |
| NFKB1 | rs3774959 |
| REL | rs2231884  rs7608910 |
| STAT4 | rs1517352 |
| TNFAIP3 | rs6920220 |
| UCN2 | rs3197999 |

^*^SNPs: single nucleotide polymorphisms

1. Jostins L, Ripke S, Weersma RK, Duerr RH, McGovern DP, et al. (2012) Host-microbe interactions have shaped the genetic architecture of inflammatory bowel disease. Nature 491: 119-124.

2. Granlund A, Flatberg A, Ostvik AE, Drozdov I, Gustafsson BI, et al. (2013) Whole genome gene expression meta-analysis of inflammatory bowel disease colon mucosa demonstrates lack of major differences between Crohn's disease and ulcerative colitis. PLoS One 8: e56818.
